# Youth baseline and state pain-related injustice appraisals are associated with emotional responses of anger and sadness: An experimental study

**DOI:** 10.3389/fpain.2023.1080461

**Published:** 2023-04-21

**Authors:** Frederick Daenen, Aline Wauters, Dimitri M.L. Van Ryckeghem, Zina Trost, Tine Vervoort

**Affiliations:** ^1^ Department of Experimental-Clinical and Health Psychology, Ghent University, Ghent, Belgium; ^2^ Department of Clinical Psychological Science, Maastricht University, Maastricht, Netherlands; ^3^ Department of Behavioural and Cognitive Sciences, University of Luxembourg, Eschsur-Alzette, Luxembourg; ^4^ Department of Physical Medicine and Rehabilitation, Virginia Commonwealth University, Richmond, VA, United States

**Keywords:** pain, injustice, children, anger, sadness, attention bias

## Abstract

**Background:**

Youth pain-related injustice appraisals are associated with adverse functioning; however, mechanisms by which injustice appraisals exert their impact have yet to be elucidated. Adult injustice literature suggests anger, sadness, and attention bias to anger (AB) as potential mechanisms. This study examined the effects of injustice appraisals in a healthy youth sample by applying a justice violation manipulation. We hypothesized the justice violation condition to lead to worse pain outcomes with effects mediated by anger, sadness, and AB as compared to the control condition. We further explored associations between both baseline and state injustice appraisals and anger, sadness, and AB across conditions.

**Methods:**

A 2 × 2 time by condition design was used to test hypotheses. 133 healthy youth aged 9–16 years old completed two cold pressor tasks (CPTs). In the experimental (i.e., justice violation) group, participants were initially told to complete one CPT, but were told afterwards to perform it again due to experimenter negligence. In the control group, no justice violation occurred. Baseline injustice appraisals and pain catastrophizing were assessed with the Injustice Experience Questionnaire and Pain Catastrophizing Scale for Children; state outcomes (i.e., injustice, catastrophizing, anger, sadness) were assessed after CPTs. AB was indexed using a dot-probe task.

**Results:**

Findings indicated no effects of the justice violation on pain outcomes or associated mechanisms, nor on injustice appraisals, suggesting manipulation failure. However, across conditions, baseline and state injustice appraisals were positively associated with anger and sadness, but not with AB.

**Conclusions:**

Despite the experimental justice violation failing to elicit differential injustice appraisals across conditions, the current study supports both anger and sadness as key emotional responses associated with pain-related injustice appraisals in a healthy youth sample.

## 1. Introduction

Pain-related injustice appraisals have been conceptualized as a set of cognitions comprising attributions of blame, magnitude of loss, and irreparability of loss (1). Research has shown pain-related injustice appraisals to be associated with worse pain outcomes in both chronic and acute pain populations of adults (2–6). Although research has found pain-related injustice appraisals to be consistently associated with pain catastrophizing, the adverse effects of injustice appraisals remain robust after controlling for pain catastrophizing (4, 7, 8). Recent research has extended these findings to both healthy and pediatric pain populations (9, 10), attesting to the potential importance of this construct as a target for clinical intervention. However, current evidence in youth samples remains cross-sectional, limiting causal interpretations of the effect of injustice appraisals. Further, associated mechanisms through which injustice appraisals may exert their effect in the context of pain have yet to be examined in youth samples. It is crucial to extend this line of research to youth samples: Potential interventions targeting maladaptive pain appraisals may be particularly beneficial in youth, with the concept of fairness both developing and being especially salient early (11–14), and moral reasoning skills being linked with cognitive development (15–18).

The broader adult injustice literature as well as literature in the context of pain has identified two primary emotional responses associated with injustice: anger and sadness (19, 20). First, anger is a commonly investigated emotional response, with adult literature showing anger to be associated with injustice appraisals outside the context of pain studies (21, 22), as well as in chronic pain samples (23–25). Anger has further been found to mediate the association between injustice appraisals and pain intensity in adults (3). Relatedly, attention bias to anger (AB) has been found to be associated with higher anger (26–28) as well as higher pain-related injustice appraisals (29), suggesting AB may be another way through which injustice appraisals exert their impact. In addition, sadness has been demonstrated to be a second common emotional response associated with perceived injustice in adults outside the context of pain (19, 21, 30), and has further been found to be associated with perceived injustice within the context of pain in adults as well as in youth (24, 31, 32). Taken together, feelings of anger, attention bias to anger, and sadness, are conceptualized in this study as potentially underlying mechanisms of the impact that injustice appraisals may exert on pain outcomes.

The current study aimed to experimentally examine the effects of pain-related injustice appraisals in a healthy youth sample by applying a justice violation manipulation in the experimental group. We hypothesized the justice violation (compared to a control condition) to lead to worse experimental pain outcomes (i.e., higher pain intensity and lower pain tolerance) as well as to higher levels of anger and sadness, and higher AB to anger. We further hypothesized heightened anger and sadness, as well as AB, to mediate the effects of the justice violation on pain outcomes. We further explored the associations between pain-related injustice appraisals and anger, sadness, and AB across both conditions. Injustice appraisals were assessed on both baseline level, at the start of the experiment, and state level, after performing an experimental pain induction task.

## 2. Method

### 2.1. Study design

The discussed study results are part of a larger study protocol comprising 2 independent research aims: (1) to examine the effect of a memory reframing intervention on pain-related outcomes, and (2) to examine the effect of pain-related injustice appraisals on pain outcomes in a sample of healthy youth. The current study reports findings exclusively related to study aim 2. Procedures relevant to research aim 1 are not expected to interfere with the current results. Both study aims used an experimental and a control group; the control group was shared between both study aims, while both experimental groups were independent of each other. The study design for study aim 2 was a 2 × 2 factorial between-subjects design, with two different groups (experimental and control) both undergoing a Cold Pressor Task (CPT) twice and reporting on identical outcomes each time. Ethical approval was obtained from the Ethics Committee of the Faculty of Psychology and Educational Sciences, Ghent University (Belgium). This study was preregistered before starting data collection; the preregistration can be viewed at Open Science Framework (OSF): https://osf.io/b7ztr.

### 2.2. Participants

Participants were recruited *via* social media calls and flyers that were distributed at schools and local sports and youth groups. An e-mail address was listed so that interested parents and their children could contact the research coordinator. When parents and their children indicated interest, they were telephoned to explain what participation entailed, and to screen for inclusion and exclusion criteria. Inclusion criteria entailed: (1) age between 9 and 16 years old, and (2) Dutch-speaking. Exclusion criteria entailed: (1) having a developmental disorder (e.g., ADHD or ASD), (2) experiencing chronic pain (i.e., defined as pain lasting longer than 3 months), and (3) having previously taken part in a research study involving pain. Power analyses to obtain.80 power indicated a target sample size of 128 participants for study aim 2 (MorePower 6.0), based on 2 × 2 mixed-design ANOVA analyses to detect medium effects (*η*² = .06) at standard.05 alpha error probability. We slightly overrecruited (initial *N* = 134) to account for participants who would not attend or not complete the full study. Participants were randomly assigned to one of both equally sized conditions (i.e., the experimental group or the control group) using block randomization. The random number list used to create these blocks was developed using the web applications available at http://random.org. Based on this predetermined list and the order in which participants arrived at the lab, participants were allocated to either the experimental or the control group. As such, participants were blind to the group allocation—the researchers were not. One participant was excluded from the analyses as the experimental manipulation was not performed correctly. The final sample for study aim 2 concerned 133 participants (51.88% girls), with a mean age of 12.52 years (*SD *= 2.29). Participants were reimbursed €25 for their participation, and were entered into a raffle to win an iPad among the participants taking place after data collection was completed.

### 2.3. Apparatus

A Cold Pressor Task (CPT) was used to experimentally induce pain. This method has previously been used successfully in youth samples (31, 33–35). Particularly, a cold pressor apparatus maintained the water temperature in a water tank at 10°C, a predetermined, painful level, and circulated water with a pump preventing heat building up around the hand or arm. The CPT was performed twice: For the first CPT, participants submersed their right hand; For the second CPT, participants submersed their left hand, to avoid effects from having performed the CPT once already carrying over to the second CPT. Prior to the CPT, participants submersed their hand in a bucket with room temperature water for two minutes to standardize their hand temperature (33, 36). During CPT performance, participants were asked to submerse their hand for as long as they could stand it, up to the wrist, with a blinded time limit of four minutes.

### 2.4. Measures

#### 2.4.1. Baseline and state pain-related injustice appraisals

Pain-related injustice appraisals (further: “injustice appraisals”) were assessed at both baseline and state level. On a baseline level, injustice appraisals were assessed at the start of the study using the Injustice Experience Questionnaire (IEQ) (1). The IEQ comprises 12 items describing injustice appraisals in the context of pain (e.g., “it all seems so unfair”), rated on a 5-point Likert scale ranging from 0 (“never”) to 4 (“all the time”). Items are summed to create a total score ranging from 0 to 48 with higher scores indicating higher levels of injustice appraisals. As the original instructions relate to specific injury, instructions were adapted to daily life instead (9, 10). While 2 subscales were identified in the IEQ by the original authors and others (1, 8, 37), i.e., “severity/irreparability of loss” and “blame/unfairness”, both subscales generally strongly correlate, and often interpreted as a unitary construct. This approach was also suggested by the original authors, and is further supported by exploratory factor analysis similarly suggesting a unitary factor solution in the current study. Good reliability and validity of the Dutch translation of the IEQ has previously been demonstrated in a youth sample (9). The IEQ showed good reliability in the current study (*α* = .87).

At a state level, injustice appraisals were assessed after each CPT completion using a 4-item scale, comprising items that assess to what extent participants (1) thought it was unfair they had to perform the CPT, (2) thought it was someone else's fault they had to perform the CPT, (3) thought they missed out on other fun activities due to the CPT, and (4) thought their pain during the CPT was not taken seriously. The first 3 items were drawn from the IEQ and adapted to be situation-specific; these items were selected due to their feasibility to be adapted to a situational context, with the first item reflecting a general feeling of injustice, the second item reflecting blame, and the third item reflecting loss of activities. Other items (e.g., “my life will never be the same”) were deemed difficult to adapt to the specific CPT context. The fourth item was added based on recent research showing invalidation to be a core theme in pain-related injustice (38). Items were rated from 0 (“not at all”) to 10 (“very much so”) and summed to create a total score ranging from 0 to 40 with higher scores indicating higher levels of state injustice appraisals. The state injustice appraisals scale showed average to good reliability in the current study (*α*_CPT1_ = .60, *α*_CPT2_ = .78).

#### 2.4.2. Baseline and state pain catastrophizing

Baseline youth pain catastrophizing was assessed at the start of the study using the Pain Catastrophizing Scale for Children (PCS-C) (39). The PCS-C is a child-adjusted version of the Pain Catastrophizing Scale (40) and comprises 13 items rated on a 5-point Likert scale ranging from 0 (=“not at all”) to 4 (=“extremely”), assessing pain catastrophizing thoughts through rumination, magnification, and feelings of helplessness related to pain. Items are summed to create a total score ranging from 0 to 52, with higher scores indicating higher levels of pain catastrophizing thoughts. Good reliability and validity of the Dutch translation of this questionnaire have previously been demonstrated in youth samples (9, 39). The PCS-C showed good reliability in the current study (*α* = .87).

State pain catastrophizing about the CPT was assessed after each CPT completion using the 3-item state version of the PCS-C (41, 42), which consists of one item for each of the three PCS-C subscales. The 3-item version was chosen over the 6-item version, with research showing a high correlation between both versions and the shorter form providing greater utility (43). All 3 items are rated on an 11-point numerical rating scale ranging from 0 (“not at all”) to 10 (“very much”). Items are summed to create a total score ranging from 0 to 30, with higher scores indicating higher levels of pain catastrophizing thoughts. The state pain catastrophizing scale has previously been used successfully to examine catastrophizing in youth in response to experimentally induced pain (35, 44), and showed adequate to good reliability in the current study (*α*_CPT1_ = .77, *α*_CPT2_ = .81).

#### 2.4.3. Pain intensity and pain tolerance

Pain intensity was assessed immediately after CPT completion using the Faces Pain Scale Revised (FPS-R) (45, 46), a self-report measure to assess state pain intensity in youth samples. The FPS-R consists of one item showing 6 faces expressing varying levels of pain, ranging from “no pain” (coded as 0) to “the most pain possible” (coded as 5). After each CPT completion participants were asked to indicate how much pain they experienced during the CPT by circling the corresponding face. The FPS-R has previously been used successfully in Dutch-speaking youth samples (35, 47). Pain tolerance was assessed during each CPT performance. Particularly, participants were told to keep their hand in the water for as long as they could stand it, with a to them unknown maximum limit of four minutes. The CPT time was recorded in seconds as an index of pain tolerance, with a maximum of 240 s.

#### 2.4.4. Anger and sadness

Pain-related anger and sadness in response to performing the CPT were assessed immediately after CPT completion with two single-item scales; participants were asked to rate the extent to which they experienced anger or sadness while performing the CPT on scales from 0 (“not at all”) to 10 (“extremely”).

#### 2.4.5. Attention bias to anger

##### 2.4.5.1. Stimulus set

Attention bias to anger (AB) was assessed with a dot-probe task and indexed as a reaction time. The stimulus set consisted of 20 images of 10 children (5 boys, 5 girls) with two images of each child: one with an angry facial expression and one with a neutral facial expression. Four extra test images (i.e., two image pairs of angry and neutral expressions) were included at the beginning of the task to get acquainted with the task. Images were selected from the Radboud Faces Database by withholding those with highest assigned anger scores (48), and have been used previously to assess AB in youth (49).

##### 2.4.5.2 Dot-probe task

The dot-probe task was programmed and presented using the Millisecond Software package INQUISIT; reaction times were measured with millisecond accuracy (50). Participants sat at about 60 cm from the computer screen. To maximize the participant's understanding of the task, instructions were provided verbally as well as on the screen prior to commencing the task. During the task, for each trial, participants were instructed to focus on a white fixation cross displayed for 500 ms in the middle of the screen against a black background. Then, a pair of images (angry and neutral expressions) were shown for a further 500 ms, respectively above and below the center of the screen. Immediately afterwards, a probe (i.e., a white rectangle of 1 cm × 1 cm) was displayed at the location of one of both images. Participants had to indicate the location of the probe as fast as possible by pressing either the Q key for the top location or the P key for the bottom location on a QWERTY keyboard. Trials ended with a pause of 400 ms, either after a response or after 2,500 ms; if the wrong response was given by the participant, an error message was displayed for 200 ms. These test trials made up 160 of a total of 178 trials. The other 18 trials were digit trials: To maintain the attention of the participants on the middle of the screen, these trials did not show an image pair, but instead showed a random digit between 1 and 9 for 150 ms at the center of the screen; participants then had to enter the correct digit as a response (51, 52). The order of the 178 trials was randomized across participants. For the test trials, the % of congruent and incongruent trials was equal and all stimuli were presented equally at each location for each trial type. Prior to starting the full set of 178 trials, participants completed a practice phase of 20 trials. In case participants experienced difficulties with completing the practice trials, the researcher clarified the instructions until the participant understood; the researcher then restarted the practice phase until the participant could complete practice trials accurately.

To index AB, trials were recorded as congruent when the probe location matched the location of the anger image; trials were recorded as incongruent when the probe location matched the location of the neutral image. Mean reaction time scores for both congruent and incongruent trials were calculated per participant. Outliers were defined as responses faster than 200 ms or slower than 2000ms and were not taken into account in the analyses. AB scores were then computed by subtracting the mean congruent trial reaction time from the mean incongruent reaction time, with positive scores reflecting a faster reaction time on trials where the probe was presented at the location of anger faces; higher scores reflect higher AB to anger.

### 2.5. Procedure

#### 2.5.1. Experimental manipulation

The experimental condition concerned an injustice manipulation modeled after the justice violation manipulation in an adult sample reported by Trost and colleagues (53). Following Trost and colleagues, all participants completed two CPTs. However, whereas participants in the control condition were informed about this beforehand, participants in the experimental condition were only told about one CPT initially. They were told later to complete the CPT a second time due to experimenter negligence. In line with previous qualitative findings in healthy youth suggesting that blame and subsequent feelings of lack of control are key themes through which youth may perceive pain to be “undeserved” (38), we expected the focus on experimenter negligence to lead to heightened youth injustice appraisals in the experimental group. Additionally, a novel element was introduced to the manipulation in an attempt to further maximize the resulting injustice appraisals: Specifically, upon arrival to the lab, participants in the experimental condition were falsely informed that the experiment would end with a Virtual Reality (VR) task, and that they would be able to select one of several options to their preference as a fun VR game. To increase the credibility of this aspect of the manipulation, a fake VR set was presented to the participant. To check whether participants were actually interested in doing the VR task, they were asked to rate how much fun they thought it would be on a scale from 0 to 10, with participants scoring 8.10 on average (SD* *= 1.40). Participants in the control condition were not informed about a VR task. In line with the procedure by Trost and colleagues, after having completed the first CPT, participants in the experimental condition were informed that they had to perform it again due to experimenter negligence regarding the research equipment. Experimenters were instructed to convey this information to the participants with a sense of carelessness and to avoid sounding apologetic. Furthermore, participants were then also informed that they would not be able to perform the VR task anymore, as the second CPT would take up more time than was initially planned.

#### 2.5.2. General procedure

Testing took place between June and October 2021. In this study, only the procedure relevant to study aim 2 is described; however, as the control group was shared with study aim 1, participants in the control group, and accordingly also in the experimental group, also completed some tasks that are only relevant to study aim 1; these are described summarily (see OSF for a full description). Participants and their parent arrived at the lab and completed consent forms before the parent left again until the end of the experiment. Instructions differed between both conditions, as described above. Next, participants started by completing a set of demographic and baseline questionnaires (Limesurvey 2.0). Afterwards, participants were informed they would shortly start the CPT and rated several state items relevant to the larger study protocol concerning the upcoming CPT. The CPT was then performed a first time, as described in the Apparatus section. Participants then completed the state questions concerning the CPT they had just completed. Subsequently, they performed tasks relevant to study aim 1: a memory retrieval task, two computer tasks, i.e., a dot-probe task to assess attention bias to pain, as well as an attention switching task, and a state questionnaire about their current emotional state (see OSF for more details). Afterwards, the experimental manipulation described above took place. Participants then completed the CPT a second time, identically to the first time but with the left instead of the right hand, as well as identical state questions before and after CPT completion. Afterwards, participants performed a dot-probe task on a computer to assess attention bias to anger information. Participants and their parents were then debriefed.

### 2.6. Statistical analyses^1^

Statistical analyses are split between preregistered analyses, i.e., the analyses performed precisely as preregistered, and exploratory analyses, i.e., analyses which were not preregistered in detail and were conducted only after having already performed the confirmatory analyses. Missing data was omitted pairwise from the analyses.

#### 2.6.1. Preregistered analyses

To test whether the experimental group would score higher on state injustice appraisals, pain intensity, feelings of anger and sadness, and lower on pain tolerance after the injustice manipulation (i.e., relating to CTP2) than the control group, we performed five Time (CPT1, CPT2) by Condition (experimental, control) 2 × 2 mixed design ANOVA analyses, i.e., one for each outcome. As several key variables were not normally distributed, non-parametric independent samples Mann-Whitney U tests were conducted to test for differences on CPT2 outcome scores (i.e., state injustice appraisals, pain intensity, pain tolerance, and feelings of anger and sadness) between conditions, to ensure convergent results with the 2 × 2 mixed design ANOVA analyses. To test our hypothesis that the experimental group would score higher on AB for anger information than the control group, we performed an independent samples T-test. Finally, to examine whether the potential mechanisms [anger, AB (to anger), and sadness] mediated the association of the condition level on either pain intensity or pain tolerance after CPT2, we performed mediation analyses using a bootstrapping method with 5,000 resamples through the JASP program (54). Weights were calculated for each path of the model. First, the total effect of condition on the outcome measure consists of the direct effect of condition on the outcome measure plus the indirect effect of condition on the outcome measure through the potential mechanism. This indirect effect ab is partialled out by calculating the effect of condition on the potential mechanism, and the effect of the potential mechanism on the outcome measure. Following Zhao & colleagues (55), we consider the presence of an indirect effect, with the 95% CI not containing zero, as the only condition to establish mediation.

#### 2.6.2. Exploratory analyses

Scores for the four state injustice items were explored individually to take note of potentially different scoring patterns among the four injustice themes represented by the items (i.e., general unfairness, blame, loss, and invalidation). As we will discuss in further detail below, our injustice manipulation failed to lead to meaningful group differences, which did not allow for our preregistered analyses to provide useful information regarding the relation between injustice appraisals and either the potential mechanisms or the pain outcomes. As such, we chose to conduct post-hoc exploratory analyses on the associations between (both state and baseline) injustice appraisals and the other study variables across the full sample. While these analyses are purely cross-sectional and cannot answer the study hypotheses described in the introduction, the results may still shed light on how injustice appraisals relate to pain outcomes and emotional reactions to pain experiences in child samples, a population in which such data as of yet remains scarce. More specifically, associations between (total) state injustice appraisals and other state measures (pain intensity, pain tolerance, anger, and sadness) were explored through correlation analyses, controlling for state pain catastrophizing, age, sex, and condition. As many key study variables were not normally distributed, Kendall Tau-b correlation analyses were used as a non-parametric alternative to Pearson correlation analyses. Kendall Tau-b correlation was chosen over Spearman correlation due to its stronger robustness for dealing with outliers (56–58). We further examined the relationship between baseline injustice appraisals and state outcomes (i.e., state injustice appraisals, pain intensity, pain tolerance, anger, sadness, and AB) through Kendall Tau-b correlation analyses, similarly controlling for baseline pain catastrophizing, age, sex, and condition.

## 3. Results

### 3.1. Descriptives

Valid cases, means, SDs, minimum and maximum values of study variables are reported in Table 1. Baseline injustice appraisals and baseline pain catastrophizing correlated strongly, in line with previous research (*r *= .67, *p *< .001). Independent samples Mann–Whitney *U*-tests revealed no significant sex differences in study variables (all *p *> .14), apart from CPT2 state injustice appraisals with boys scoring higher than girls [*U*(68,65) = 1,762.5, *p *= .03]. Older participants reported lower baseline injustice appraisals (*r *= −.23, *p *= .008), in line with previous findings on the association between age and injustice appraisals in a non-clinical youth sample (9). Participants reported means of 2.49 (CPT 1, SD* *= 1.19) and 2.50 (CPT2, SD* *= 1.19) on a scale from 0 to 5 for pain intensity, suggesting both CPT procedures were successful in inducing a reasonable amount of pain. Older participants showed higher pain tolerance than younger participants for both CPTs (*r*_CPT1_* = *.53, *p *< .001; *r*_CPT2_* *= .51, *p *< .001). For both CPTs, participants reported very low levels of injustice (scale range 0–40, *M*_CPT1_* *= 3.02, *M*_CPT2_ = 2.41), sadness (scale range 0–10, *M*_CPT1_* *= 0.42, *M*_CPT2_ = 0.37), and anger (scale range 0–10, *M*_CPT1_* *= 0.34, *M*_CPT2_ = 0.29). Mann-Whitney U-tests were conducted to compare the CPT2 state scores between the experimental and control conditions: We found no differences for any variables, suggesting the justice violation manipulation failed to work (all *p *> .25).

**Table 1:** Descriptive statistics of key study variables.

	Age	IEQC	PCSC	AB	Pain 1	Tol 1	Inj 1	Cat 1	Sad 1	Anger 2	Pain 2	Tol 2	Inj 2	Cat 2	Sad 2	Anger 2
*N*	133	132	132	133	133	133	133	133	133	133	133	133	133	133	133	133
*M*	12.52	12.22	15.42	−2.442	2.50	140.54	3.02	8.96	0.42	0.34	2.50	123.17	2.41	8.62	0.37	0.29
SD	2.29	7.69	8.17	26.80	1.19	99.80	4.31	6.08	1.20	1.04	1.19	103.25	4.87	6.47	1.15	1.20
Min	9	0	4	−97.06	0	11	0	0	0	0	0	6	0	0	0	0
Max	16	41	45	85.72	5	240	25	29	6	6	5	240	34	29	7	9

*Note*. PCSC = trait pain catastrophizing; Pain = pain intensity, Tol = pain tolerance; Inj = state injustice appraisals; Cat = state pain catastrophizing; Ang = anger. Numbers 1 or 2 are added to denote variables assessed in relation to respectively CPT1 or CPT2.

### 3.2. Confirmatory analyses

#### 3.2.1. Manipulation impact on CPT outcomes

2 × 2 mixed design analyses with factors Time (CPT1, CPT2) and Condition (Experimental, Control) were performed for the outcomes (1) state injustice appraisals, (2) pain intensity, (3) pain tolerance, (4) anger, and (5) sadness. For state pain-related injustice appraisals, analyses showed no main effects of condition [*F*(1, 131) = 0.33, *p* = .56] or time [*F*(1,131) = 3.32, *p* = .07]. There was no interaction between the effects of condition and time [*F*(1,131) = 0.01, *p* = .93]. For pain intensity, analyses showed no main effects of condition [*F*(1,131) = 1.01, *p* = .32] or time [*F*(1,131) = 0.01, *p* = .93]. There was no interaction between the effects of condition and time [*F*(1,131) = 0.99, *p* = .32]. For pain tolerance, analyses showed a main effect of time [*F*(1,131) = 14.85, *p* < .001, *ω*² = 0.01] with participants scoring a higher pain tolerance at CPT1 than at CPT2; but not of condition [*F*(1,131) = 0.01, *p* = .94]. There was no interaction between the effects of condition and time [*F*(1,131) = 3.58, *p* = .06]. For feelings of anger, analyses showed no main effects of condition [*F*(1,131) = 0.61, *p* = .43] or time [*F*(1,131) = 0.25, *p* = .61]. There was no interaction between the effects of condition and time [*F*(1,131) = 0.03, *p* = .87]. For feelings of sadness, analyses showed no main effects of condition [*F*(1,131) = 0.32, *p* = .57] or time [*F*(1,131) = 0.91, *p* = .34]. There was also no interaction between the effects of condition and time [*F*(1,131) = 1.48, *p* = .23].

#### 3.2.2. Manipulation impact on attention bias to anger

An independent samples T-test showed no significant difference in AB scores between the experimental and control condition [*t*(131) = 0.37, *p *= .72].

#### 3.2.3. Mediation analyses

We performed mediation analyses to assess whether anger, AB to anger, or sadness mediated associations between the group condition and pain outcomes after CPT2 (i.e., pain intensity and pain tolerance). Standardized estimates are reported.

For anger, mediation analyses showed no direct effect of condition on pain intensity (*β* = −0.20, *SD *= 0.17, 95% CI = [−0.52, 0.13] or on pain tolerance (*β* = 0.08, SD* *= 0.17, 95% CI = [−0.26, 0.41], nor an indirect effect of anger in the association between condition and pain intensity (*β* = −0.03, *SD *= 0.04, 95% CI = [−0.11, 0.06] or pain tolerance (*β* = 0.01, SD* *= 0.02, 95% CI = [−0.03, 0.06].

For AB to anger, mediation analyses showed no direct effect of condition on pain intensity (*β* = −0.22, SD* *= 0.17, 95% CI = [−0.56, 0.12] or on pain tolerance (*β* = 0.10, SD* *= 0.17, 95% CI = [−0.25, 0.43], nor an indirect effect of AB in the association between condition and pain intensity (*β* = −0.00, SD* *= 0.01, 95% CI = [−0.03, 0.02] or pain tolerance (*β* = 0.00, SD* *= 0.00, 95% CI = [−0.01, 0.01].

For sadness, mediation analyses showed no direct effect of condition on pain intensity (*β* = −0.18, SD* *= 0.17, 95% CI = [−0.50, 0.15] or on pain tolerance (*β* = 0.06, SD* *= 0.17, 95% CI = [−0.27, 0.40], nor an indirect effect of sadness in the association between condition and pain intensity (*β* = −0.04, SD* *= 0.05, 95% CI = [−0.14, 0.05] or pain tolerance (*β* = 0.03, SD* *= 0.04, 95% CI = [−0.04, 0.10].

### 3.3. Exploratory analyses

Additional analyses independent of the manipulation were conducted to examine how state and baseline injustice appraisals were associated with outcomes across both conditions, rather than on a group level.

#### 3.3.1. Associations between state injustice appraisals and outcomes across conditions

As the scale to examine state injustice appraisals was novel to this study, we first examined whether the four state injustice appraisal items showed differential scoring patterns. All four state injustice appraisal items scored similarly low across both conditions (all *M *< 1 on a range of 0–10; see Table 2). As the items were not normally distributed, Mann-Whitney U tests were conducted to assess differences on item-level between conditions: no differences were detected (all *p *> .09).

**Table 2:** Descriptive statistics of state injustice appraisal items for control (C) and experimental (E) conditions.

	Inj 1.1		Inj 1.2		Inj 1.3		Inj 1.4		Inj 2.1		Inj 2.2		Inj 2.3		Inj 2.4	
	C	E	C	E	C	E	C	E	C	E	C	E	C	E	C	E
*N*	68	65	68	65	68	65	68	65	68	65	68	65	68	65	68	65
*M*	0.84	0.68	0.40	0.26	0.97	0.91	1.00	0.97	0.34	0.58	0.43	0.40	0.96	0.78	0.91	0.42
SD	1.44	1.46	1.25	0.99	2.14	1.57	1.95	1.71	1.19	1.61	1.26	1.41	2.08	1.78	1.87	1.03
IQR	1	1	0	0	1	1	1	1	0	0	0	0	1	1	1	0
Shapiro-Wilk	0.64^***^	0.53^***^	0.37^***^	0.29^***^	0.53^***^	0.65^***^	0.59^***^	0.64^***^	0.30^***^	0.42^***^	0.38^***^	0.32^***^	0.52^***^	0.50^***^	0.56^***^	0.47^***^

*Note*. The injustice items are grouped by CPT (CPT 1 or 2) and assessed to what extent children 1) thought it was unfair they had to perform the CPT, 2) thought it was someone else's fault they had to perform the CPT, 3) thought they missed out on other fun activities due to the CPT, and 4) thought their pain during the CPT was not taken seriously. ***p < .001.

Associations between state injustice appraisals and outcomes are reported in Table 3. Across both conditions, after CPT1 completion, Kendall Tau-b correlation analyses showed state injustice appraisals to be associated with higher sadness (*τ*_b_ = .29, *p *< .001), and higher anger (*τ*_b_ = .22, *p *= .003), but not pain intensity (*τ*_b_ = .09, *p *= .20) or pain tolerance (*τ*_b_ = −.11, *p *= .10). After controlling for state pain catastrophizing, age, sex, and condition, both associations with higher sadness (*τ*_b_ = .24, *p *< .001) and higher anger (*τ*_b_ = .18, *p *= .003) remained significant. After CPT2 completion, state injustice appraisals were associated with higher pain intensity (*τ*_b_ = .17, *p *= .02), higher sadness (*τ*_b_ = .32, *p *< .001), and higher anger (*τ*_b_ = .32, *p *< .001), but not pain tolerance (*τ*_b_ = −.02, *p *= .75). After controlling for state pain catastrophizing, age, and sex, only the associations with higher sadness (*τ*_b_ = .28, *p *< .001) and higher anger remained significant (*τ*_b_ = .28, *p *< .001). Finally, AB was not associated with state injustice appraisals for CPT1 (*τ*_b_ = −.06, *p *= .36) or CPT2 (*r *= −.09, *p *= .15).

**Table 3:** Kendall Tau-b partial correlations.

	IEQC	Inj 1/2
Pain 1	.09	−.04
Tol 1	−.04	.05
Sad 1	.14^*^	.23^***^
Anger 1	.13^*^	.18^**^
Pain 2	.13^*^	.05
Tol 2	−.06	.07
Sad 2	.16^**^	.28^***^
Anger 2	.12^*^	.28^***^

*Note*. Kendall Tau-b partial correlations conditioned on child age, sex, condition, and respectively trait (for IEQC) or state (for Inj 1/2) pain catastrophizing. IEQC = trait injustice appraisals, Inj 1/2 = state injustice appraisals for CPT1 or CPT2.

*p < .05, **p < .01, ***p < .001.

#### 3.3.2. Associations between baseline injustice appraisals and outcomes across conditions

Associations between baseline injustice appraisals and outcomes are reported in Table 3. For CPT1, Kendall Tau-b correlation analyses showed baseline injustice appraisals to be associated with higher pain intensity (*τ*_b_ = .15, *p *= .02), lower pain tolerance (*τ*_b_ = −.17, *p *= .01), higher state injustice appraisals (*τ*_b_ = .22, *p *< .001), higher sadness (*τ*_b_ = .24, *p *< .001), and higher anger (*τ*_b_ = .19, *p *= .007). After controlling for baseline pain catastrophizing, age, sex, and condition, baseline injustice appraisals were only associated with higher state injustice appraisals (*τ*_b_ = .14, *p *= .02), higher sadness (*τ*_b_ = .14, *p *= .02), and higher anger (*τ*_b_ = .13, *p *= .03). For CPT2, Kendall Tau-b correlation analyses showed baseline injustice appraisals to be associated with higher pain intensity (*τ*_b_ = .18, *p *= .007), lower pain tolerance (*τ*_b_ = -.18, *p *= .004), higher state injustice appraisals (*τ*_b_ = .15, *p *= .03), higher sadness (*τ*_b_ = .25, *p *< .001), and higher anger (*τ*_b_ = .19, *p *= .008). After controlling for baseline pain catastrophizing, age, sex, and condition, baseline injustice appraisals were only associated with higher pain intensity (*τ*_b_ = .13, *p *= .03), higher sadness (*τ*_b_ = .16, *p *= .006), and higher anger (*τ*_b_ = .12, *p *= .04). Finally, baseline injustice appraisals were not associated with AB (*r *= .12, *p *= .16).

## 4. Discussion

The current study aimed to experimentally examine the effects of pain-related injustice appraisals in a healthy youth sample by applying a justice violation manipulation in the experimental group. We hypothesized that the experimental group would score higher on pain-related injustice appraisals, pain outcomes, as well as the hypothesized mechanisms of anger, sadness, and AB to anger, which we further hypothesized to all mediate the effect of the experimental manipulation on pain outcomes. Contrary to expectations, no differences were found between the experimental and control condition for the included state outcomes. As data inspection showed that state injustice appraisals after manipulation did not differ between groups, this was not surprising, leading us to conclude that the experimental manipulation was unsuccessful. We similarly found no support for our hypothesis that anger, AB for anger, or sadness would mediate effects of the justice violation on pain outcomes.

The experimental manipulation applied in this study was modeled on the justice violation applied in an acute adult pain sample by Trost and colleagues (53), in which participants in the experimental condition were made to complete a CPT a second time due to feigned experimenter negligence. Trost and colleagues found higher anger ratings after the manipulation in the experimental condition compared to a control condition, where no justice violation manipulation happened. Furthermore, they also found higher pain ratings among the experimental condition, but only among participants who scored high on just world beliefs, i.e., an individual's tendency to view the world as fair and predictable (59, 60). We attempted to strengthen this manipulation further by introducing an element of loss, by having participants miss out on a promised, fun VR task due to experimenter negligence. Yet, the experimental manipulation failed, with no differences between conditions for any study variables. We tentatively offer five potential causes. First, the voluntary nature of participation guarantees participants a sense of control and safety—participants in psychology studies might be quick to suspect deception and are made aware that they always have the option to quit the study without consequence. Second, the justice violation might have been too weak for participants to be sufficiently affected; indeed, when communicating the justice violation, some participants afterwards noted they felt sorry for the researchers rather than having experienced feelings of injustice. Third, participants were healthy youth experiencing acute pain; while not exclusively, most research on pain-related injustice appraisals involves chronic pain patients. Indeed, associations between injustice appraisals and worse pain outcomes were weaker in a healthy youth sample compared to a pediatric pain sample (9). Recent qualitative work has found youth injustice appraisals in response to chronic pain manifest very differently from feelings of unfairness in response to acute pain, suggesting injustice perceptions in a chronic pain context do not translate to an acute pain context (38). Fourth, it is possible that, in line with findings of Trost and colleagues in an adult sample (53), effects of the manipulation may have been dependent on participants' personality traits such as their just world beliefs, or sensitivity to injustice. Initial aims of this study included exploring the associations between personality traits and injustice appraisals, however, scales used to assess personality traits showed insufficient reliability to be used in the analyses. As research outside the context of pain has found personality traits to be associated with a higher sensitivity to injustice (61), future research should aim to assess whether pain-related injustice appraisals and associated responses manifest differently based on personality traits. Fifth, we must note that, despite these reservations, Trost and colleagues succeeded in applying a similar experimental manipulation in adults. As such, another potential cause of our lack of findings may be that the manipulation does not adequately translate from adult to youth samples, as youth may be more obedient by nature than adults, and may have been more likely to simply defer to an authority figure.

Taken together, these considerations may suggest that attempting to apply an experimental manipulation centered around a justice violation condition is not feasible in the context of pain in samples of healthy children. Future research attempting to study state pain-related injustice appraisals is likely to run into the same issues as described above, with the aforementioned qualitative work suggesting that the nature of injustice appraisals differs greatly in clinical populations. Nevertheless, we should not rule out the possibility for future research to successfully apply a novel method of experimentally manipulation pain-related injustice appraisals in healthy samples. While the study by Trost and colleagues (53) is, to the best of our knowledge, the only prior study to apply such a manipulation, further inspiration may be drawn from manipulations outside the context of pain. In adult samples, injustice perceptions have been manipulated successfully by modifying supervisor feedback (62, 63), or by applying vignette studies (64). In child samples, injustice perceptions have been manipulated successfully by arbitrarily assigning privilege to students (65). Future research may adapt such manipulations in the context of pain, e.g., by giving arbitrary feedback based on a painful task performance, or by arbitrarily assigning one group of participants an easier task and making the other group aware of this. However, it is important to note that these manipulations may not translate to pain-related injustice, especially given the aforementioned differential nature of injustice between healthy and clinical groups, which mainly centers around feelings of (long-lasting) stigmatization and epistemic injustice (38). As such, rather than attempting to optimize a manipulation for healthy youth samples, it may be preferable to attempt to design a novel manipulation targeted at clinical samples.

As the experimental manipulation failed, we could not adequately test our preregistered, causal hypotheses. However, after establishing that our experimental manipulation failed, we conducted additional exploratory cross-sectional analyses across groups to examine the relationships between state as well as baseline injustice appraisals and state outcomes (i.e., pain intensity, pain tolerance, and feelings of anger and sadness) as well as AB for anger. Findings indicated significant associations between state as well as baseline injustice appraisals and higher feelings of both anger and sadness, strengthened by their robustness after controlling for pain catastrophizing. While these cross-sectional results cannot inform our causal preregistered hypotheses, they do attest to similar associations between injustice appraisals and both anger and sadness as found in adult samples (3, 23–25, 31, 32). This study extends these findings from adult to youth samples, suggesting that anger and sadness are key emotional responses associated with pain-related injustice appraisals in youth as well, hence potentially pointing to a key role for emotion regulation when targeting injustice appraisals. Against expectations, we found neither baseline or state injustice appraisals to be associated with AB for anger, contradicting previous findings by Baert and colleagues who found injustice appraisals to be associated with higher AB for anger in an adult sample using an eye tracking paradigm (29). Notably, AB for anger was also not associated with self-reported feelings of anger either in the current study, contradicting findings in the anger literature (26–28). The absence of associations with AB for anger might possibly be explained by the use of dot-probe methodology to assess AB, with previous research noting discrepancies in findings on AB literature and suggesting the presentation timing of faces to be a possible cause (66). Future research on AB may benefit from using an eye tracking paradigm less prone to the influence of the exact presentation timing such as in a dot-probe task.

Several study limitations should be noted. First, caution should be exerted when interpreting these results, as scores for key study variables state injustice appraisals, anger, and sadness were all low, indicating that participants generally did not perceive having to complete the pain task as unfair, nor did they experience notable anger or sadness in response to the pain task. This is in line with a previous observational study involving youth taking part in a CPT and reporting on emotional responses (31); our attempt to introduce more variability in these scores by applying an experimental injustice manipulation failed. Experimental manipulation failure raises questions as to whether pain-related injustice can indeed successfully be induced within experimental lab settings and how this could -within future studies- ideally be done. A second limitation relates to the measures used to assess both baseline and state pain-related injustice appraisals. Baseline injustice appraisals were assessed using the IEQ, which, while validated across both adult and youth as well as both chronic and acute pain sample, was initially developed for adults in the context of persistent pain after injury. Adjustments made for youth or for an acute pain context are limited and more research is needed into pain-related injustice measures in youth. A third limitation concerns the inclusion of a healthy sample. In line with previous findings in a cross-sectional study comparing the associations between injustice appraisals and pain-related outcomes in both healthy and clinical child samples (9), it is likely that the found associations would be more pronounced in a chronic pain sample. Replication of observed findings in a pediatric pain sample is needed. Fourth, due to feasibility concerns, the current study applied power analyses to obtain.80 power for detecting medium effect sizes—as such, this study was not adequately powered to detect small effect sizes. Nevertheless, the clear failure of the experimental manipulation likely excluded that possibility as well, with no statistical test results for difference between groups trending towards significance. Fifth, to standardize hand temperature before submersing the hand in the box with cold water, participants first submersed their hands in a box with water of room temperature. However, room temperature variations may have affected the water temperature, leading to inconsistent hand temperatures prior to the submersing of the hand in the cold water. Finally, sixth, exclusion criteria as applied in the current study were not stringently in line with the exclusion criteria as suggested by von Baeyer et al. (34), as participants were not screened for a history of cardiovascular disorder, fainting, seizures, frostbite, or Reynaud's phenomenon. To ensure maximal standardization and safety of the experimental procedure across studies, future research should more explicitly include the suggested criteria.

In conclusion, the current study failed to apply an experimental justice violation manipulation to induce variation in pain-related injustice appraisals, rendering the examination of the preregistered hypotheses concerning group differences impossible. Multiple suggestions for the manipulation failure are discussed, shedding light on the potential future application of justice violation manipulation in child samples. Furthermore, while the *a priori* hypotheses could not be examined, and despite the limitations described above, post-hoc exploratory analyses found the associations between pain-related injustice appraisals and both anger and sadness to be in line with results from adult samples, even after controlling for pain catastrophizing, age, and sex. Future research should attempt to assess the preregistered causal hypotheses in a pediatric chronic pain context.

## Data Availability

The raw data supporting the conclusions of this article will be made available by the authors, without undue reservation.

## Footnotes

^1^In line with the preregistration, personality components and trait anger were assessed for exploratory purposes using the Hierarchical Personality Inventory for Children short form (HiPIC-30) (67) and Anger Expression Scale for Children (AESC) (68) respectively. However, these measures are not reported on in this study, as the questionnaires showed poor reliability. Similarly, state measures assessed prior to CPT completion are not reported on in this study due to very low reliability (for state injustice appraisals assessed prior to CPT completion: α_CPT1_=.53, α_CPT2_=.61).

## References

[1] Sullivan MJL Adams H Horan S Maher D Boland D Gross R. The role of perceived injustice in the experience of chronic pain and disability: scale development and validation. J Occup Rehabil. (2008) 18:249–61. 10.1007/s10926-008-9140-5 18536983

[2] Carriere JS Pimentel SD Yakobov E Edwards RR. A systematic review of the association between perceived injustice and pain-related outcomes in individuals with musculoskeletal pain. Pain Medicine. (2020) 21:1449–63. 10.1093/PM/PNAA088 32377686

[3] Scott W Trost Z Bernier E Sullivan MJL. Anger differentially mediates the relationship between perceived injustice and chronic pain outcomes. Pain. (2013) 154:1691–8. 10.1016/j.pain.2013.05.015 23707294

[4] Sullivan MJL Scott W Trost Z. Perceived injustice: a risk factor for problematic pain outcomes. Clinical Journal of Pain. (2012) 28:484–8. 10.1097/AJP.0b013e3182527d13 22673480

[5] Yakobov E Scott W Stanish WD Tanzer M Dunbar M Richardson G Reductions in perceived injustice are associated with reductions in disability and depressive symptoms after total knee arthroplasty. Clinical Journal of Pain. (2018) 34:415–20. 10.1097/AJP.0000000000000551 28877144

[6] Lynch J Fox S D’Alton P Gaynor K. A systematic review and meta-analysis of the association between perceived injustice and depression. J Pain. (2021) 22:643–54. 10.1016/j.jpain.2020.12.009 33465504

[7] Sullivan MJL Adams H Martel MO Scott W Wideman T. Catastrophizing and perceived injustice: risk factors for the transition to chronicity after whiplash injury. Spine. (2011) 36:S244–9. 10.1097/BRS.0b013e3182387fed 22020619

[8] Yakobov E Scott W Stanish W Dunbar M Richardson G Sullivan M. The role of perceived injustice in the prediction of pain and function after total knee arthroplasty. Pain. (2014) 155:2040–6. 10.1016/j.pain.2014.07.007 25064836

[9] Daenen F McParland J Baert F Miller MM Hirsh AT Vervoort T. Child pain-related injustice appraisals mediate the relationship between just-world beliefs and pain-related functioning. Eur J Pain. (2021) 25:757–73. 10.1002/ejp.1707 33259693

[10] Miller MM Scott EL Trost Z Hirsh AT. Perceived injustice is associated with pain and functional outcomes in children and adolescents with chronic pain: a preliminary examination. J Pain. (2016) 17:1217–26. 10.1016/j.jpain.2016.08.002 27555426

[11] Fehr E Bernhard H Rockenbach B. Egalitarianism in young children. Nature. (2008) 454:1079–83. 10.1038/nature07155 18756249

[12] Jose PE. Just-world reasoning in children’s immanent justice judgments. Child Dev. (1990) 61:1024–33. 10.1111/j.1467-8624.1990.tb02839.x 2209175

[13] Murnighan JK Saxon MS. Ultimatum bargaining by children and adults. J Econ Psychol. (1998) 19:415–45. 10.1016/S0167-4870(98)00017-8

[14] Sutter M. Outcomes versus intentions: on the nature of fair behavior and its development with age. J Econ Psychol. (2007) 28:69–78. 10.1016/j.joep.2006.09.001

[15] Enright RD Bjerstedt A Enright WF Levy VM Lapsley DK Buss RR Distributive justice development: cross-cultural, contextual, and longitudinal evaluations. Child Dev. (1984) 55:1737. 10.2307/1129921

[16] Güroğlu B van den Bos W Crone EA. Fairness considerations: increasing understanding of intentionality during adolescence. J Exp Child Psychol. (2009) 104:398–409. 10.1016/j.jecp.2009.07.002 19674756

[17] Jacobs JE Klaczynski PA. The development of judgment and decision making during childhood and adolescence. Curr Dir Psychol Sci. (2002) 11:145–9. 10.1111/1467-8721.00188

[18] Kohlberg L. Moral stages and moralization. A cognitive developmental approach. J Stud Educ Dev. (1982) 5:33–51. 10.1080/02103702.1982.10821935

[19] Mikula G Scherer KR Athenstaedt U. The role of injustice in the elicitation of differential emotional reactions. Pers Soc Psychol Bull. (1998) 24:769–83. 10.1177/0146167298247009

[20] Miller DT. Disrespect and the experience of injustice. Annu Rev Psychol. (2001) 52:527–53. 10.1146/annurev.psych.52.1.527 11148316

[21] Khan AK Quratulain S Crawshaw JR. The mediating role of discrete emotions in the relationship between injustice and counterproductive work behaviors: a study in Pakistan. J Bus Psychol. (2013) 28:49–61. 10.1007/S10869-012-9269-2

[22] Barclay LJ Skarlicki DP Pugh SD. Exploring the role of emotions in injustice perceptions and retaliation. J Appl Psychol. (2005) 90:629–43. 10.1037/0021-9010.90.4.629 16060783

[23] Sturgeon JA Carriere JS Kao MCJ Rico T Darnall BD Mackey SC. Social disruption mediates the relationship between perceived injustice and anger in chronic pain: a collaborative health outcomes information registry study. Ann Behav Med. (2016) 50:802–12. 10.1007/s12160-016-9808-6 27325314 PMC5127748

[24] Yakobov E Suso-Ribera C Vrinceanu T Adams H Sullivan MJ. Trait perceived injustice is associated with pain intensity and pain behavior in participants undergoing an experimental pain induction procedure. Journal of Pain. (2019) 20:592–9. 10.1016/j.jpain.2018.11.007 30503859

[25] Carriere JS Sturgeon JA Yakobov E Kao MC MacKey SC Darnall BD. The impact of perceived injustice on pain-related outcomes: a combined model examining the mediating roles of pain acceptance and anger in a chronic pain sample. Clin J Pain. (2018) 34:739. 10.1097/AJP.0000000000000602 29485535 PMC6424103

[26] Maoz K Adler AB Bliese PD Sipos ML Quartana PJ Bar-Haim Y. Attention and interpretation processes and trait anger experience, expression, and control. Cogn Emot. (2017) 31:1453–64. 10.1080/02699931.2016.1231663/SUPPL_FILE/PCEM_A_1231663_SM4404.ZIP 27653208

[27] Cohen DJ Eckhardt CI Schagat KD. Attention allocation and habituation to anger-related stimuli during a visual search task. Aggress Behav. (1998) 24:399–409. 10.1002/(SICI)1098-2337(1998)24:6<399::AID-AB1>3.0.CO;2-I

[28] Owen JM. Transdiagnostic cognitive processes in high trait anger. Clin Psychol Rev. (2011) 31:193–202. 10.1016/J.CPR.2010.10.003 21094569

[29] Baert F van Ryckeghem D Sanchez-Lopez A Miller MM Hirsh AT Trost Z The impact of maternal child- and self-oriented pain-related injustice appraisals upon maternal attention to child pain, attention to anger, and pain-attending behavior. Br J Pain. (2022) 16:303–16. 10.1177/20494637211057092 35646345 PMC9136993

[30] Chory RM Horan SM Carton ST Houser ML. Toward a further understanding of Students’ emotional responses to classroom injustice. Commun Educ. (2014) 63:41–62. 10.1080/03634523.2013.837496

[31] Daenen F Baert F van Ryckeghem D Trost Z Vervoort T. The relationship between child pain-related injustice appraisals and child pain-related attention, anger, sadness, and maternal pain-attending behavior: a preliminary investigation. Manuscript in Preparation. (2022).

[32] Kennedy L Dunstan DA. Confirmatory factor analysis of the injustice experience questionnaire in an Australian compensable population. J Occup Rehabil. (2014) 24:385–92. 10.1007/S10926-013-9462-9/TABLES/3 23979003

[33] Birnie KA Petter M Boerner KE Noel M Chambers CT. Contemporary use of the cold pressor task in pediatric pain research: a systematic review of methods. J Pain. (2012) 13:817–26. 10.1016/j.jpain.2012.06.005 22846592

[34] von Baeyer CL Piira T Chambers CT Trapanotto M Zeltzer LK. Guidelines for the cold pressor task as an experimental pain stimulus for use with children. J Pain. (2005) 6:218–27. 10.1016/j.jpain.2005.01.349 15820909

[35] Wauters A Noel M van Ryckeghem DML Sanchez-Lopez A Vervoort T. Parental (non-)pain attending verbalizations moderate the relationship between child attention and memory bias for pain. Eur J Pain. (2020) 24:1797–811. 10.1002/ejp.1627 32618062

[36] Vervoort T Trost Z Van Ryckeghem DML. Children’s selective attention to pain and avoidance behaviour: the role of child and parental catastrophizing about pain. Pain. (2013) 154:1979–88. 10.1016/j.pain.2013.05.052 23792243

[37] Rodero B Luciano J V. Montero-Marín J Casanueva B Palacin JC Gili M López del Hoyo Y, Serrano-Blanco A Garcia-Campayo J. Perceived injustice in fibromyalgia: psychometric characteristics of the injustice experience questionnaire and relationship with pain catastrophising and pain acceptance. J Psychosom Res (2012) 73:86–91. 10.1016/j.jpsychores.2012.05.011 22789409

[38] Daenen F Baert F McParland J Trost Z Vervoort T. Child appraisals of injustice in the context of pain: an interpretative phenomenological analysis. Manuscript in Preparation. (2022). 10.1002/ejp.2154 37381085

[39] Crombez G Bijttebier P Eccleston C Mascagni T Mertens G Goubert L The child version of the pain catastrophizing scale (PCS-C): a preliminary validation. Pain. (2003) 104:639–46. 10.1016/S0304-3959(03)00121-0 12927636

[40] Sullivan MJL Bishop SR Pivik J. The pain catastrophizing scale: development and validation. Psychol Assess. (1995) 7:524–32. 10.1037/1040-3590.7.4.524

[41] Vervoort T Goubert L Eccleston C Verhoeven K De Clercq A Buysse A The effects of parental presence upon the facial expression of pain: the moderating role of child pain catastrophizing. Pain. (2008) 138:277–85. 10.1016/j.pain.2007.12.013 18243557

[42] Vervoort T Caes L Trost Z Sullivan M Vangronsveld K Goubert L. Social modulation of facial pain display in high-catastrophizing children: an observational study in schoolchildren and their parents. Pain. (2011) 152:1591–9. 10.1016/j.pain.2011.02.048 21459512

[43] Durand H Birnie KA Noel M Vervoort T Goubert L Boerner KE State versus trait: validating state assessment of child and parental catastrophic thinking about children’s acute pain. J Pain. (2017) 18:385–95. 10.1016/j.jpain.2016.11.012 27919776

[44] Heathcote LC Lau JYF Mueller SC Eccleston C Fox E Bosmans M Child attention to pain and pain tolerance are dependent upon anxiety and attention control: an eye-tracking study. Eur J Pain. (2017) 21:250–63. 10.1002/ejp.920 27463940

[45] Hicks CL Von Baeyer CL Spafford PA Van Korlaar I Goodenough B. The faces pain scale—revised: toward a common metric in pediatric pain measurement. Pain. (2001) 93:173–83. 10.1016/S0304-3959(01)00314-1 11427329

[46] Stinson JN Kavanagh T Yamada J Gill N Stevens B. Systematic review of the psychometric properties, interpretability and feasibility of self-report pain intensity measures for use in clinical trials in children and adolescents. Pain. (2006) 125:143–57. 10.1016/j.pain.2006.05.006 16777328

[47] Brands MMMG Purperhart H Deckers-Kocken JM. A pilot study of yoga treatment in children with functional abdominal pain and irritable bowel syndrome. Complement Ther Med. (2011) 19:109–14. 10.1016/J.CTIM.2011.05.004 21641514

[48] Langner O Dotsch R Bijlstra G Wigboldus DHJ Hawk ST van Knippenberg A. Presentation and validation of the radboud faces database. Cogn Emot. (2010) 24:1377–88. 10.1080/02699930903485076

[49] Mastorakos T Scott KL. Attention biases and social-emotional development in preschool-aged children who have been exposed to domestic violence. Child Abuse Negl. (2019) 89:78–86. 10.1016/J.CHIABU.2019.01.001 30639972

[50] De Clercq A Crombez G Buysse A Roeyers H. A simple and sensitive method to measure timing accuracy. *Behavior Research Methods*. Instruments, and Computers. (2003) 35:109–15. 10.3758/BF03195502 12723785

[51] Van Ryckeghem DML Crombez G Goubert L De Houwer J Onraedt T Van Damme S. The predictive value of attentional bias towards pain-related information in chronic pain patients: a diary study. Pain. (2013) 154:468–75. 10.1016/j.pain.2012.12.008 23375161

[52] Vervoort T Trost Z Prkachin KM Mueller SC. Attentional processing of other’s facial display of pain: an eye tracking study. Pain. (2013) 154:836–44. 10.1016/j.pain.2013.02.017 23561271

[53] Trost Z Scott W Lange JM Manganelli L Bernier E Sullivan MJ. An experimental investigation of the effect of a justice violation on pain experience and expression among individuals with high and low just world beliefs. Eur J Pain. (2014) 18:415–23. 10.1002/j.1532-2149.2013.00375.x 23918230

[54] JASP Team. JASP. (2022). Available at: https://jasp-stats.org/ (Accessed July 15, 2022).

[55] Zhao X Lynch JG Chen Q. Reconsidering baron and kenny: myths and truths about mediation analysis. J Consum Res. (2010) 37:197–206. 10.1086/651257

[56] Croux C Dehon C. Influence functions of the spearman and kendall correlation measures. Stat Methods Appt. (2010) 19:497–515. 10.1007/s10260-010-0142-z

[57] Hollander M Wolfe DA Chicken E. Nonparametric statistical methods. Hoboken, New Jersey: Wiley (2015). 10.1002/9781119196037

[58] Khamis H. Measures of association: how to choose? J Diagn Med Sonogr. (2008) 24:155–62. 10.1177/8756479308317006

[59] Lerner MJ. The belief in a just world. Boston, MA: Springer US. (1980). p. 9–30. 10.1007/978-1-4899-0448-5_2

[60] Dalbert C. Belief in a just world. In: Leary MR Hoyle RH, editors. Handbook of individual differences in social behavior. New York, NY: Guilford Press (2009). p. 288–97. https://books.google.com/books?hl=en&lr=&id=67xcAgAAQBAJ&oi=fnd&pg=PA288&dq=dalbert+2009&ots=DVgEJYz1vO&sig=JwsNq5bsHHUGjdh8Px-7UDsQfI4 (Accessed July 22, 2022).

[61] Žitný P Halama P. Self-esteem, locus of control, and personality traits as predictors of sensitivity to injustice. Stud Psychol (Bratisl). (2011) 53:27–40. https://www.researchgate.net/profile/Peter-Halama/publication/286392962_Self-esteem_locus_of_control_and_personality_traits_as_predictors_ofsensitivity_to_in_justice/links/57501a1f08ae4eed2740b8eb/Self-esteem-locus-of-control-and-personality-traits-as-pre (Accessed June 24, 2022).

[62] Long EC Christian MS. Mindfulness buffers retaliatory responses to injustice: a regulatory approach. J Appl Psychol. (2015) 100:1409–22. 10.1037/apl0000019 25751750

[63] Reb J Goldman BM Kray LJ Cropanzano R. Different wrongs, different remedies? Reactions to organizational remedies after procedural and interactional injustice. Pers Psychol. (2006) 59:31–64. 10.1111/j.1744-6570.2006.00773.x

[64] Umphress EE Simmons AL Folger R Ren R Bobocel R. Observer reactions to interpersonal injustice: the roles of perpetrator intent and victim perception. J Organ Behav. (2013) 34:327–49. 10.1002/job.1801

[65] Pretsch J Ehrhardt N Engl L Risch B Roth J Schumacher S Injustice in school and Students’ emotions, well-being, and behavior: a longitudinal study. Soc Justice Res. (2016) 29:119–38. 10.1007/s11211-015-0234-x

[66] Cooper RM Langton SRH. Attentional bias to angry faces using the dot-probe task? It depends when you look for it. Behav Res Ther. (2006) 44:1321–9. 10.1016/J.BRAT.2005.10.004 16321361

[67] Vollrath ME Hampson SE Torgersen S. Constructing a short form of the hierarchical personality inventory for children (HiPIC): the HiPIC-30. Personal Ment Health. (2016) 10:152–65. 10.1002/PMH.1334 27120426

[68] Steele RG Legerski JP Nelson TD Phipps S. The anger expression scale for children: initial validation among healthy children and children with cancer. J Pediatr Psychol. (2009) 34:51–62. 10.1093/jpepsy/jsn054 18556672 PMC2722119

